# From Cerebrospinal Fluid to Blood Draw: Plasma p-Tau217 as a Non-Invasive Biomarker for Alzheimer’s Disease: A Fagan Nomogram-Based Meta-Analytic Study

**DOI:** 10.1007/s12035-026-05864-2

**Published:** 2026-05-04

**Authors:** Nesma M. Gebril, Abdelrahman M. Elettreby, Abeer H. Younis, Mostafa Hossam El Din Moawad, Afaf M. Hafez, Gamal El Sayed, Kariman M. Abdelrahman, Sarah M. El-Kot

**Affiliations:** 1 National Center for Social and Criminological Research, Giza, 3755153 Egypt; 2 Faculty of Medicine, Mansoura University, Mansoura, Egypt; 3 National Institute of Oceanography and Fisheries (NIOF), Alexandria, 21556 Egypt; 4 Alexandria Main University Hospital, Alexandria, Egypt; 5 Faculty of Medicine, Suez Canal University, Ismailia, Egypt; 6 Environmental Studies Department, Institute of Graduate Studies and Research, Alexandria University, Alexandria, 21526 Egypt; 7 Biochemistry Department, Faculty of Science, Alexandria University, Alexandria, 21568 Egypt; 8 Wastewater Lab, Baheria Water and Waste Company, Damanhur 107, Baheria, Egypt; 9 Biochemistry and Nutrition Department, Girls’ College, Ain Shams University, Cairo, Egypt

**Keywords:** Alzheimer’s disease, Plasma p-tau217, Biomarker, Diagnosis, Meta-analysis

## Abstract

Alzheimer’s disease (AD) is the leading cause of dementia worldwide and is pathologically defined by amyloid-β and tau accumulation. Current diagnostic methods, such as PET imaging and cerebrospinal fluid (CSF) assays, are accurate but invasive, costly, and limited in accessibility. Plasma phosphorylated tau at threonine 217 (p-tau217) has emerged as a promising blood-based biomarker, but evidence from individual studies remains heterogeneous. We conducted a systematic review and meta-analysis to evaluate the diagnostic performance of plasma p-tau217 for AD. Following PRISMA guidelines, PubMed, Scopus, and Web of Science were searched up to July 2025. Eligible studies included clinical or biomarker-defined AD cohorts that reported plasma p-tau217 accuracy against amyloid or tau positivity or clinical diagnosis. Data on sensitivity, specificity, likelihood ratios, and diagnostic odds ratio (DOR) were extracted. Study quality was assessed using QUADAS-2. Pooled estimates were calculated using a Bayesian bivariate model, and heterogeneity was explored with meta-regression and subgroup analyses. Twenty-seven studies including 19,652 participants were analyzed. Plasma p-tau217 demonstrated high diagnostic accuracy for biomarker-defined AD, with pooled sensitivity of 85.4% (95% posterior intervals [PI]: 81.4–88.7), specificity of 88.0% (95% PI: 85.1–90.6), positive likelihood ratio (PLR) 7.13, negative likelihood ratio (NLR) 0.167, and DOR 42.7. Performance was consistent across amyloid PET and CSF reference standards. Subgroup analyses showed robust accuracy for amyloid positivity (sensitivity 87.3%, specificity 85.5%), tau positivity (sensitivity 84.9%, specificity 93.8%), and clinical AD diagnosis (sensitivity 72.9%, specificity 89.5%). Plasma p-tau217 consistently outperformed other blood biomarkers and correlated with cognitive decline, frailty, and behavioral impairment. Risk of bias was generally low, with no major publication bias detected. This meta-analysis indicates that plasma p-tau217 demonstrates promising diagnostic accuracy for detecting AD pathology across biomarker-defined reference standards. However, heterogeneity across assays, populations, and reference definitions, along with the use of optimized cut-offs in some studies and the limited power of publication-bias assessments, warrant cautious interpretation. Plasma p-tau217 appears well suited as a triage biomarker to guide confirmatory testing, but further large, prospectively designed studies with standardized assays and externally validated thresholds are needed before widespread clinical implementation.

**Supplementary Information:**

The online version contains supplementary material available at 10.1007/s12035-026-05864-2.

## Introduction

Alzheimer’s disease (AD) is the predominant cause of dementia globally, presently impacting over 55 million individuals, with prevalence anticipated to quadruple by 2050 [1, 2]. The condition is marked by advancing cognitive deterioration and functional deficits, constituting a significant societal and economic burden [3]. Neuropathologically, AD is characterized by extracellular amyloid-β plaques and intracellular neurofibrillary tangles comprised of hyperphosphorylated tau protein [4, 5]. Tau pathology exhibits a stronger correlation with disease severity and cognitive loss, underscoring its significance as a biomarker [6].

In-vitro biomarkers have revolutionized the diagnosis of AD. Cerebrospinal fluid (CSF) analyses of Aβ42, total tau, and phosphorylated tau, along with amyloid and tau positron emission tomography (PET), are regarded as reference standards for identifying AD pathology [7, 8]. These markers support the National Institute on Aging-Alzheimer’s Association (NIA-AA) AT(N) framework, which physiologically characterizes AD through amyloid, tau, and neurodegeneration [9]. Although PET imaging is precise, it is costly and not broadly accessible, whereas lumbar puncture for CSF collection is invasive and less tolerable for several people [10, 11]. These constraints hinder widespread clinical application, particularly in primary care and resource-constrained environments.

Consequently, blood-based biomarkers have emerged as a viable alternative. Progress in ultrasensitive techniques enables accurate quantification of circulating proteins at minimal concentrations [12]. Plasma phosphorylated tau at threonine 217 (p-tau217) has demonstrated significant efficacy, surpassing other blood-based biomarkers like p-tau181 and Aβ42/40 ratios in differentiating AD from non-AD [13, 14]. Plasma p-tau217 exhibits a robust correlation with both CSF and PET measurements of amyloid and tau pathology, with numerous studies indicating diagnostic accuracies beyond 90% in differentiating AD from other dementias [13–15].

Notwithstanding these promising results, observed performance differs among populations, tests, and reference standards. Individual investigations frequently have limited sample sizes, and methodological diversity has obstructed agreement on the therapeutic value of plasma p-tau217. A thorough review and meta-analysis is necessary to consolidate the existing evidence, deliver reliable aggregated estimates of diagnostic accuracy, and investigate causes of heterogeneity among research.

## Methods

### Search Strategy

This systematic and meta-analysis review was conducted in accordance with the PRISMA guidelines [16]. A comprehensive literature search was performed in PubMed, Scopus, and Web of Science databases up to July 2025. The search combined keywords and MeSH terms related to “AD” “Plasma” and “p-tau217”. Boolean operators (“and”, “or”) were applied to maximize sensitivity. Reference lists of eligible studies and relevant reviews were also screened manually to identify additional articles. No language restrictions were applied. The search strategy was formulated as follows: ("AD" or " Alzheimer’s" or "AD") and ("Plasma" or "Serum" or "Blood") and ("p-tau217" or " phosphorylated tau-217").

### Screening and Eligibility Criteria

All retrieved citations were imported into EndNote, and duplicates were removed. Titles and abstracts were screened, followed by full-text review of potentially eligible articles. Studies were considered eligible if they met the following criteria:

1. Study design: observational studies reporting diagnostic accuracy.
2. Population: participants with clinically diagnosed Alzheimer’s disease (AD) dementia/prodromal AD and/or participants with biomarker-confirmed AD pathology (amyloid and/or tau positivity).
3. Intervention: measurement of plasma concentration of p-tau217.
4. Comparator: none.
5. Outcomes: sensitivity, specificity, positive likelihood ratio (PLR), negative likelihood ratio (NLR) and diagnostic odds ratio (DOR).
6. Reference standard: the target condition (AD pathology/diagnosis) had to be established using an accepted reference standard, including amyloid PET and/or tau PET, CSF biomarkers consistent with AD (e.g., Aβ42 or Aβ42/40 ratio with p-tau and/or t-tau), or a clinical diagnosis of AD dementia based on established criteria (e.g., NIA-AA) when biomarker confirmation was not reported. When studies reported results against more than one reference standard, we extracted each reference-standard definition separately where possible.

Exclusion criteria included case reports, reviews, and conference abstracts without full data; studies measuring CSF p-tau217; studies comparing different tau proteins; studies comparing different diseases in the concentration of p-tau217; and studies without an independent reference standard for AD pathology/diagnosis.

### Data Extraction

Data were extracted independently by two investigators using a standardized form. Extracted variables included study identifiers (author, year, and country), sample size, demographic data (age, and sex distribution), and baseline measurements of apolipoprotein E epsilon 4 allele (APOE ε4) carriers, mini-mental state examination (MMSE) score, and plasma p-tau217. Outcome data were extracted as sensitivity, specificity, true positive (TP), false positive (FP), true negative (TN), and false negative (FN).

We also extracted the reference standard(s) used to define AD (amyloid PET, tau PET, CSF biomarkers, and/or clinical diagnostic criteria) and, when applicable, the modality used for amyloid confirmation (PET versus CSF) and the study-specific positivity thresholds. When studies reported multiple diagnostic estimates (e.g., across different thresholds, cohorts, or reference standards), each estimate was extracted separately but treated as originating from the same study. To account for within-study correlation and avoid unit-of-analysis errors, we adopted the following approach: (1) When multiple thresholds were reported, we preferentially extracted pre-specified or externally validated cut-offs; if unavailable, the primary or most clinically relevant threshold was selected, (2) When multiple cohorts within a study were independent (e.g., separate populations or validation cohorts), these were treated as separate datasets, and (3) When multiple reference standards were used (e.g., PET and CSF), results were included in subgroup analyses but not double-counted within the same pooled estimate.

This approach aligns with recommended practices for diagnostic test accuracy meta-analyses and minimizes bias from correlated observations.

### Quality Assessment

The methodological quality of included studies was assessed using the Quality Assessment of Diagnostic Accuracy Studies (QUADAS-2) tool [17]. Two reviewers independently evaluated the risk of bias across domains (patient selection, index test, reference standard, and flow/timing). Any disagreements were resolved through discussion or arbitration by a third reviewer.

### Statistical Analysis

TP, TN, FP, and FN were derived from the reported sensitivity and specificity values. The pooled diagnostic outcomes included: (1) sensitivity, (2) specificity, (3) PLR, and (4) NLR. In addition, the diagnostic odds ratio (DOR) was calculated as a single indicator of test accuracy, expressing how well the test distinguishes between individuals with and without the disease. The DOR ranges from 0 to infinity, where smaller values suggest limited accuracy and larger values indicate stronger discriminative ability [18]. Reference standards were defined a priori. For the primary meta-analysis, the target condition was AD pathology defined as positivity for amyloid or tau biomarkers (A + or T +), confirmed by PET imaging or CSF assays as reported in each study. Secondary subgroup analyses were performed according to the reference standard used (amyloid positivity, tau positivity, or a purely clinical diagnosis of AD dementia without mandatory biomarker confirmation). Within the amyloid-positivity subgroup, we additionally stratified results by modality (amyloid PET versus CSF). To visualize diagnostic performance, a bivariate summary receiver operating characteristic (SROC) curve was generated, combining sensitivity and specificity results across studies. According to Cochrane Handbook of Systematic reviews of Interventions [19], the diagnostic test accuracy meta-analysis will have heterogeneity and random effect model should be used, so we applied this. Also, pooled estimates were calculated using a Bayesian bivariate model, and heterogeneity was explored with meta-regression and subgroup analyses. Forest plots were also produced to present pooled sensitivity and specificity estimates. Deek’s funnel plot asymmetry test was applied to assess potential publication bias [20]. Finally, clinical applicability was evaluated using a Fagan nomogram [21]. All statistical analyses were performed with MetaBayesDTA [22] and Stata 17.0 [23].

## Results

**Fig. 1:**
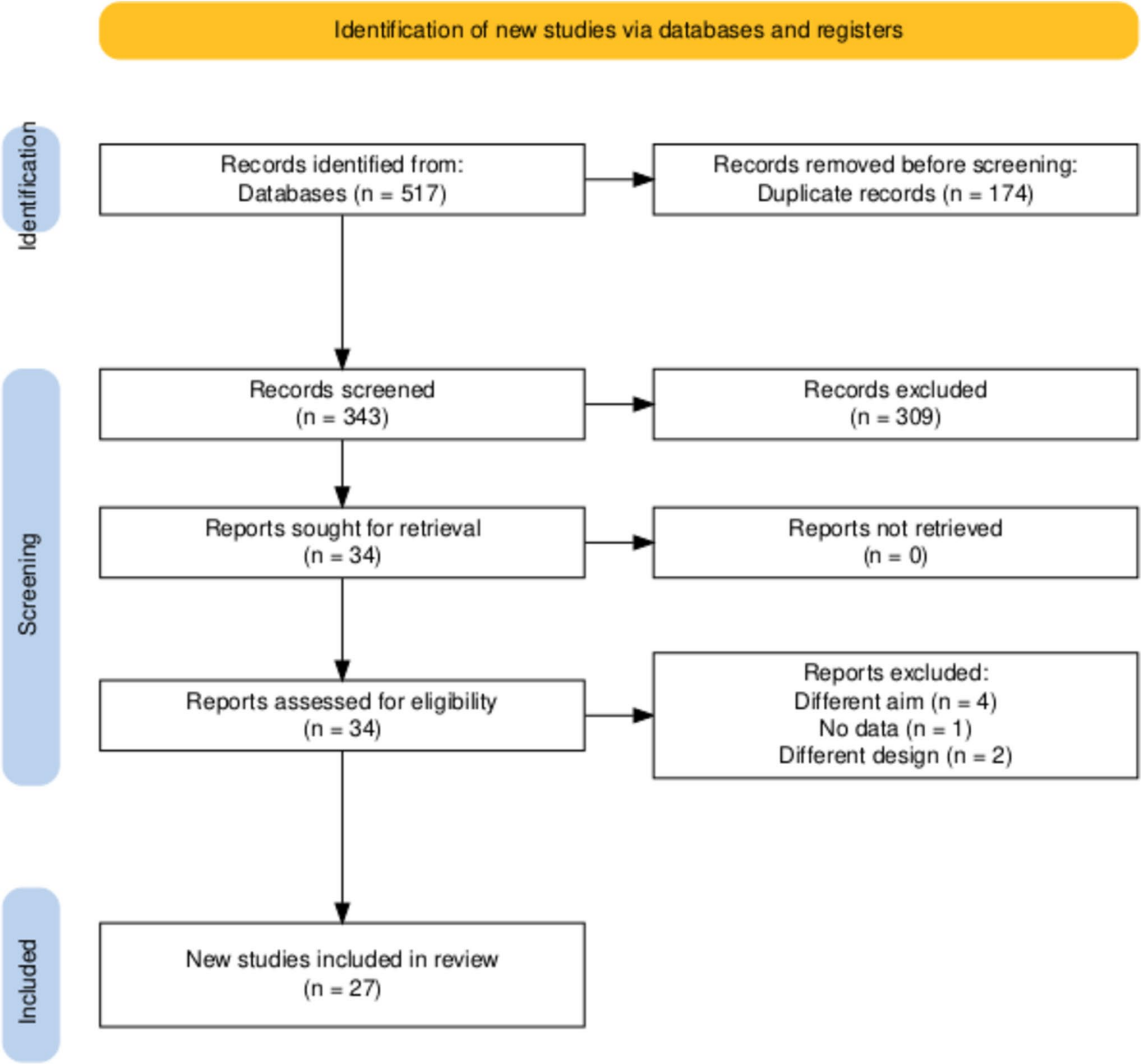
PRISMA flow diagram of searching and screening

The search yielded 517 articles, of which 174 were identified as duplicates and removed. The remaining 343 articles underwent title and abstract screening. Following this, 34 full-text articles were assessed for eligibility, resulting in the inclusion of 27 studies in the final systematic review and meta-analysis [24–50] (Fig. 1).

### Characteristics of the Patients in the Included Studies

Across the 27 studies, baseline information (reported at the group level) covered 19,652 participants. The populations spanned the clinical spectrum: cognitively unimpaired, mild cognitive impairment, and AD dementia drawn mainly from memory‐clinic cohorts with some community/research samples. Where available, the average mean age was ~ 70.6 years, 42% were men, and ~ 34% were APOE-ε4 carriers. Cognitive performance reflected mixed severity with an average mean MMSE = 24, and 12 years of education. Reported plasma p-tau217 concentrations (pg/mL) varied widely across cohorts and platforms (mean = 0.58 pg/mL), consistent with assay and case-mix differences. Studies were geographically diverse, with most data originating from the USA, followed by China, Sweden, Spain, and Italy. Overall, the dataset represents typical clinical referral populations enriched for biomarker work-ups, alongside several community/research cohorts, which together underpin the meta-analytic generalizability while introducing expected heterogeneity in age, severity, and assay distributions (Table 1).

**Table 1:** Summary and baseline of the included studies

Study ID	Group	Sample size	Country	Age (years)		Sex (Male)		APOE ε4 carriers		MMSE score		Plasma p-tau217, pg/mL	
				Mean	SD	N	%	N	%	Mean	SD	Mean	SD
Arias 2025 (TRIAD)	CU	103	Sweden	71.5	5.5	38	36.9	26	25.2	29.1	1.1	0.06	0.05
Arias 2025 (TRIAD)	MCI	30		71.7	5	12	40	14	46.7	27.9	1	0.14	0.06
Arias 2025 (TRIAD)	AD	10		68.4	8.3	6	60	7	70	24.2	4.3	0.23	0.13
Arias 2025 (Biorender2)	CU	336		66.2	11.4	156	46.4	155	46.1	28.9	1.3	0.19	0.14
Arias 2025 (Biorender2)	MCI	95		72.3	8.46	48	50.5	74	77.9	26.7	2	0.43	0.29
Arias 2025 (Biorender2)	AD	121		74.1	6.71	59	48.8	87	71.9	21.6	4.1	0.61	0.33
Ashton 2024	SPIN	195	Spain	63.5	13.8	75	38.5	81	41.5	26.4	4.19	0.977	0.766
Ashton 2024	TIAN	268	Canada	69.4	7.9	101	37.7	96	35.8	27	4.72	0.636	0.648
Ashton 2024	WRAP	323	USA	65.3	6.91	106	32.8	121	37.5	29.2	1.23	0.466	0.362
Brickman 2021	Aβ PET -	32	USA	82.16	5.19	13	41	11	34			0.15	0.14
Brickman 2021	Aβ PET +	8		84.25	4.55	3	37.5	6	75			0.39	0.18
Brickman 2021	AD	131		82.99	6.49	40	30.5	38	29			0.32	0.32
Brickman 2021	Control	169		81.01	6.31	60	36	46	27			0.18	0.17
Dakterzada 2025	AD	56	Spain	73.6	5.3	17	30.4	29	52	20.6	3.8		
Dakterzada 2025	MCI	193		74	2.2	77	39.9	72	38	25	2.9		
Dakterzada 2025	Non-AD dementia	27		74.7	7	18	66.7	5	19	23.3	7		
Ennis 2025	Aβ PET	65	USA	67.2	7.6	20	30.8					0.38	0.3
Ennis 2025	Tau-PET	70		66.2	8.7	21	30					0.41	0.36
Figdore 2024	MCI	345	USA	74.3	30.5	212	61	133	40				
Figdore 2024	Mild dementia	82		73.6	29.4	46	56	52	63.4				
Ghahremani 2025	Mild BI +	27	Canada	72.7	6.3	17	63					0.083	0.052
Ghahremani 2025	Mild BI -	74		71.8	6.6	39	52.7					0.058	0.039
Giacomucci 2025	AD	50	Italy	70.63	6.84	21	42			19.56	4.9	1.65	1.7
Giacomucci 2025	MCI Core1 +	45		70.89	8.42	18	40			26.3	2.75	0.78	0.59
Giacomucci 2025	MCI Core1-	42		68.85	8.68	18	42.8			27.49	2.12	0.19	0.11
Giacomucci 2025	SCD Core1 +	13		68.41	8.7	6	46			28.31	1.25	0.49	0.47
Giacomucci 2025	SCD Core1–	25		63.31	6.35	7	28			29.21	0.88	0.24	0.24
Groot 2022	Cohort 1 – Controls	27	Sweden	72.63	5.38	17	63	6	22.2			0.31	0.05
Groot 2022	Cohort 1 – MCI	25		71.6	5.77	10	40	19	76			0.56	0.23
Groot 2022	Cohort 2 – MCI- AD Aβ +	45		75.89	6.97	11	24.4	36	80	26.09	1.65	0.46	0.18
Groot 2022	Cohort 2 – MCI- other Aβ +	9		71.67	5.36	6	66.7	7	77.8	27.67	2.06	0.3	0.15
Groot 2022	Cohort 2 – MCI- other Aβ −	24		73.12	7.6	13	54.2	11	45.8	27	1.82	0.23	0.11
Groot 2022	Cohort 2 – Stable MCI Aβ +	18		70.39	6.7	9	50	14	77.8	27.83	1.47	0.31	0.11
Groot 2022	Cohort 2 – Stable MCI Aβ −	51		68.84	7.7	22	43.1	14	27.5	28.45	1.05	0.2	0.12
Jonaitis 2023 (WRAP)	CU	165	USA	62.94	6.06	57	35						
Kang 2025	CI	1884	Korea	71.8	8.7	699	37.1	832	44.2				
Kang 2025	CU	613		70.1	8.3	219	35.7	154	25.1				
Li 2025	AD	60	China	64.52	8.55	21	35					1.5	0.68
Li 2025	CU	150		49.54	17.04	63	42					0.24	0.12
Li 2025	Cognitively unimpaired controls	60		65.25	7.37	23	38.3					0.28	0.17
Li 2025	Mild cognitive impairment due to AD	30		67.77	6.51	11	36.6					1.07	0.49
Pandey 2025	AD	525	Peru	71.6	8	170	32.4	89	16.8			0.23	0.22
Palmqvist 2025	Malmo	337	Malmo	72	9.4	185	55.9	154	45.7	24	5	0.36	0.33
Palmqvist 2025	Gothenburg	165	GT	66	8.1	81	49	99	60	24	4.7	0.44	0.46
Palmqvist 2025	Barcelona	487	Barcelona	73	6	205	42.1	167	34.3	21	5.4	0.56	0.48
Palmqvist 2025	Brescia	230	Brescia	71	8.7	98	42.6	104	45.2	23	4.7	0.6	0.52
Palmqvist 2025	Sweden	548	Sweden	76	6.9	268	48.9	236	43.1	26	3.4	0.38	0.37
Rajbanshi 2024	Amnestic dementia	10	USA	69	8	7	70	5	50			0.79	0.7
Rajbanshi 2024	Cognitively normal	47		64	9	10	21.3	6	12.8			0.15	0.1
Rajbanshi 2024	MCI	15		70	12.3	11	73.3	1	6.7			0.2	0.1
Rousset 2024	AD dementia	138	Netherlands	65.67	8.24	70	50.7	99	71.7	21.4	4.7	1	0.82
Rousset 2024	MCI +	37		67.67	6.17	16	43.2	27	73	27.2	2	0.79	0.57
Rousset 2024	MCI −	13		67.67	6.64	4	30.8	5	38.5	27.9	2.1	0.31	0.13
Rousset 2024	SCD +	15		65.33	6.54	6	40	9	60	28.5	1.5	0.63	0.39
Rousset 2024	SCD −	89		59	7.54	40	44.9	35	39.3	28.2	2.2	0.2	0.08
Rudolph 2025	CU	314	USA	68.53	7.97	89	28.3	80	25.5			0.32	0.21
Rudolph 2025	Dementia	64		71.75	8.8	32	50	30	46.9			0.85	0.5
Rudolph 2025	MCI	213		71.32	7.37	87	40.8	77	36.2			0.49	0.39
Rudolph 2025	Other	7		70	9.63	2	28.6	0	0			0.39	0.34
Saari 2024	ALL	697	USA	76.17	4.57			203	29.2			0.45	0.31
Sarto 2025	Aβ negative	182	Spain	65	8.1	105	57.7	25	19.3	25.5	4	0.129	0.116
Sarto 2025	Aβ positive	286		67.6	7.8	131	45.8	141	56.2	22.7	4.3	0.845	0.664
Sewell 2025	ALL	632	USA	69.9	3.8	182	29	172	27			0.44	0.33
Thanapornsangsuth 2024	AD	72	Thailand	69.5	9.8	22	30.6	39	54.2			19.8	14.3
Thanapornsangsuth 2024	Non-AD	60		67.2	11.5	21	35	12	20			4.5	2.8
Thijssen 2021	Normal control	118	America	60.9	18	55	47	31	28	29	1	0.17	0.1
Thijssen 2021	MCI	99		65.5	13	55	55.6	31	34	27	2	0.29	0.3
Thijssen 2021	AD	58		65.3	10	25	43.1	37	84	19	7	0.72	0.4
Tian 2025	ALL	5149	China	70.41	5.47	2205	42.8						
Wang 2025	Aβ -	136	China	68.4	6.83	39	28.7	24	17.6	22.9	4.37	0.14	0.05
Wang 2025	Aβ +	100		68.8	7.63	31	31	38	38	16.7	6.98	0.73	0.47
Xion 2023	Non-dementia	1650	China	69.8	7.2	753	45.6	248	15.8	28.6	1.6	0.36	0.2
Xion 2023	AD dementia	145		79.1	6.1	55	37.9	33	27	25.8	3.2	0.71	0.6
Xion 2023	Non-AD dementias	62		77.8	5.9	18	29	7	13.5	25.6	3	0.43	0.3
Zhong 2024	BI-1 cohort negative	106	China	69	6.63	35	33	18	17	23.4	4.04	0.15	0.06
Zhong 2024	BI-1 cohort positive	76		69	7.59	26	44.2	29	38.2	16.9	7.18	0.7	0.46
Zhong 2024	BI-2 cohort negative	47		67	7.35	11	23.4	10	21.3	21.6	5.01	0.15	0.05
Zhong 2024	BI-2 cohort positive	31		69.2	7.87	8	25.8	13	41.9	15.5	6.58	0.75	0.48
Zhong 2024	RWCP: cohort negative	51		63.2	10.9	43	64.9	8	15.7	15.1	7.69	0.21	0.12
Zhong 2024	RWCP: cohort positive	49		67.6	10.6	19	38.8	30	61.2	11.5	7.02	0.76	0.35
Moon 2025	Aβ positive	214	USA	76.5	8	109	50.9	16.3	2.8	2.2	2.9	0.5	0.38
Moon 2025	Aβ +, CU	86		76.1	7.3	27	31.4	16.6	2.9	0.1	0.2	0.29	0.21
Moon 2025	Aβ -	196		73.6	8.9	99	55.5	16.6	2.3	0.7	1.5	0.14	0.18

Aβ: Amyloid Beta; AD: Alzheimer’s disease; BI: behavioral impairment; BI: Brain Initiative; CI: Cognitive impaired; CU: Cognitive unimpaired; MCI: Mild cognitive impairment; RWCP: Real-world clinical practice; SCD: subjective cognitive decline

### Risk of Bias Assessment

Using QUADAS-2 across the 27 studies, patient selection risk was Low in 22 studies, with 3 “some concerns” and 2 High; index-test risk was Low in 22 and High in 5, most commonly where p-tau217 cut-offs were optimized on the same dataset; reference-standard risk was Low in 23 and High in 3, typically when no independent PET/CSF biomarker was used; and flow/timing was Low in 26 and High in 1, reflecting generally short index-to-reference intervals. Applicability was largely reassuring: patient-selection applicability Low in 22 (3 High, 2 some concerns), index-test applicability Low in all 27, and reference-standard applicability Low in 22 (5 High) mirroring the few studies without biomarker confirmation. In practical terms, most evidence comes from appropriately sampled clinical cohorts with accepted reference standards; the main residual risks relate to data-driven thresholds and non-biomarker references in a minority of studies (Figures S1, and S2), (Table S1).

### Meta-Analysis

#### Overall Diagnostic Accuracy (Amyloid and/or Tau Positivity as AD Indicator)

**Fig. 2:**
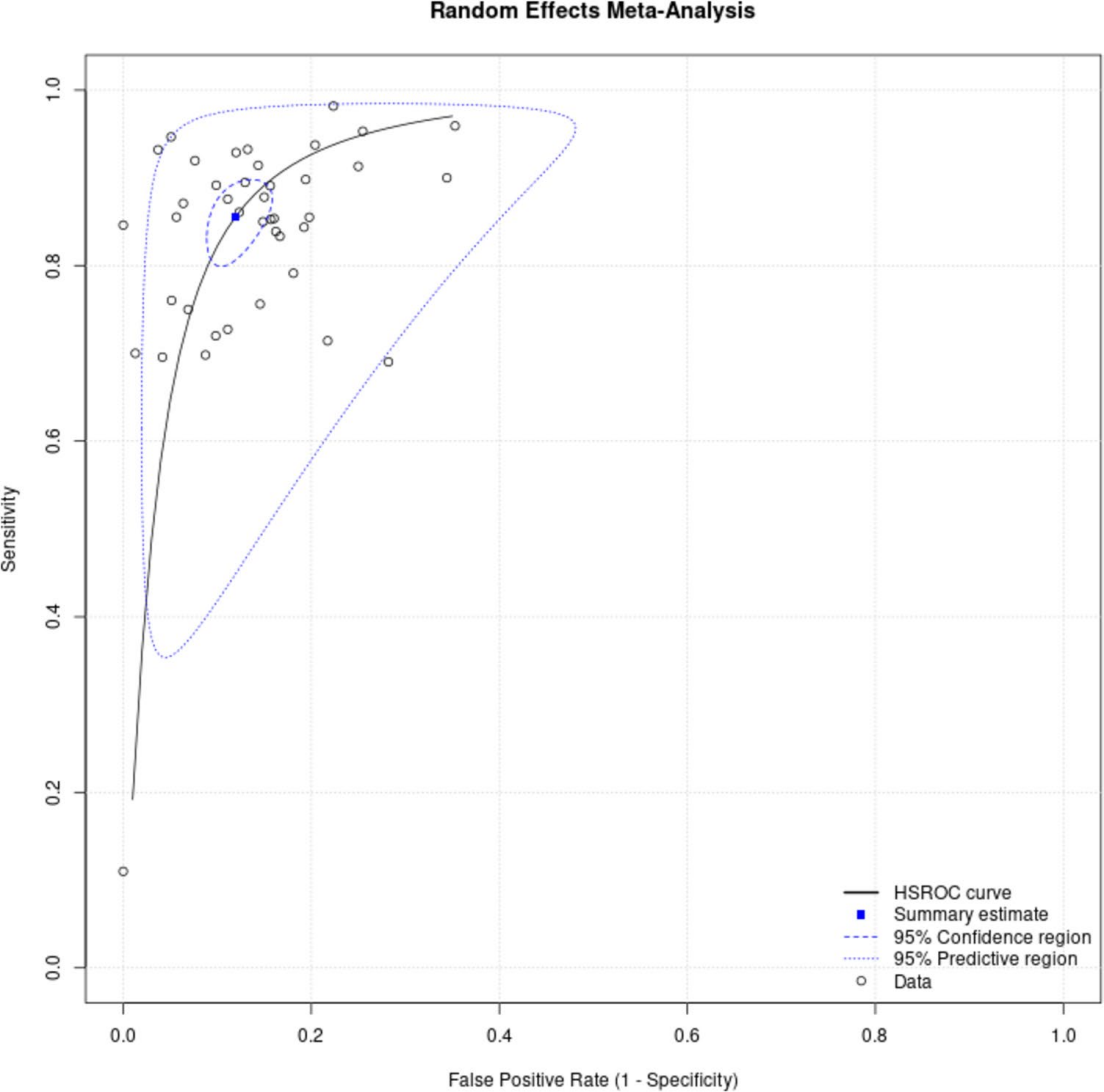
Hierarchical summary receiver operating characteristic curve showing the accuracy of p-tau217 in diagnosing AD

**Fig. 3:**
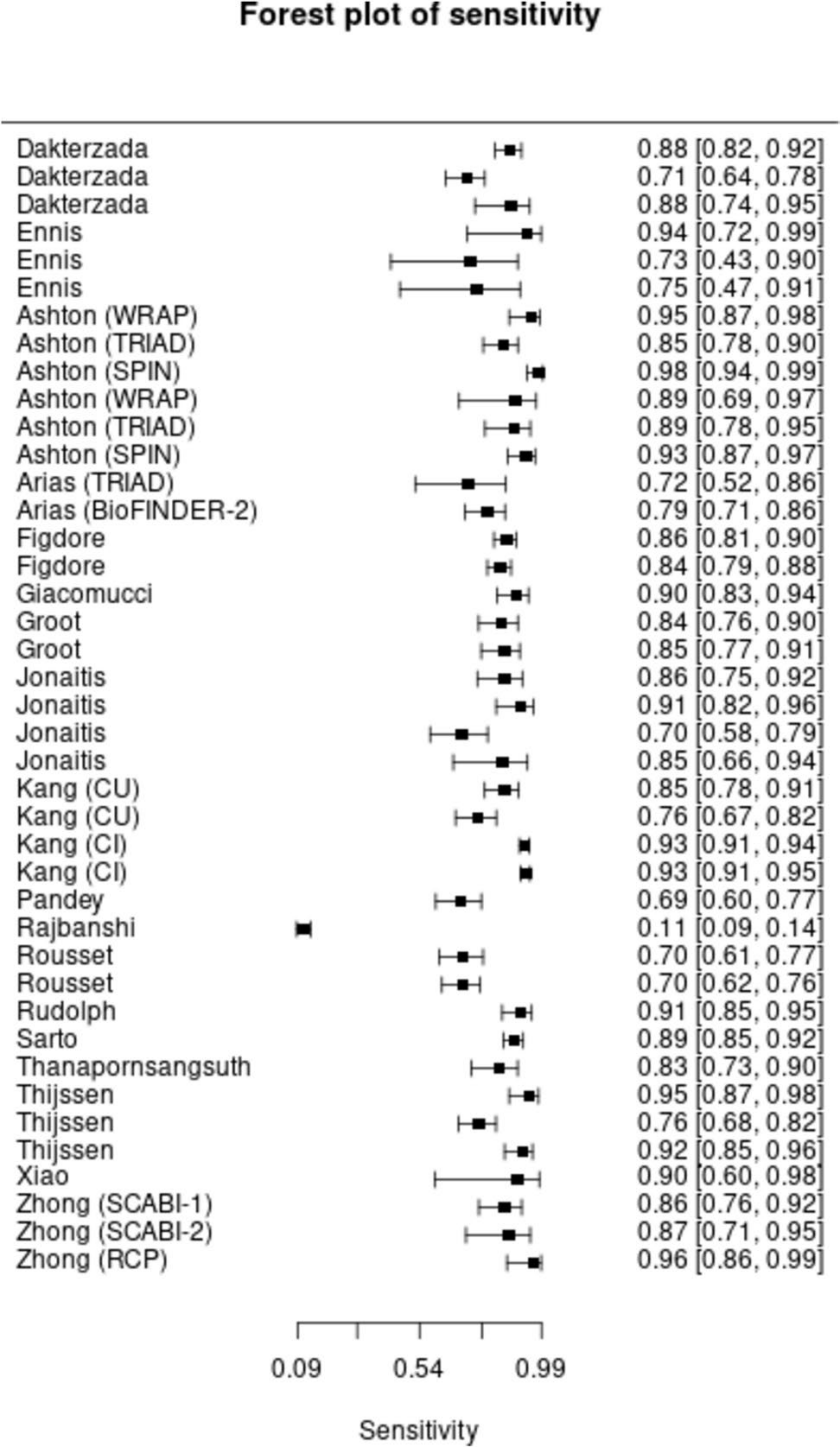
Forest Plot showing the Sensitivity of p-tau217 in diagnosing AD

**Fig. 4:**
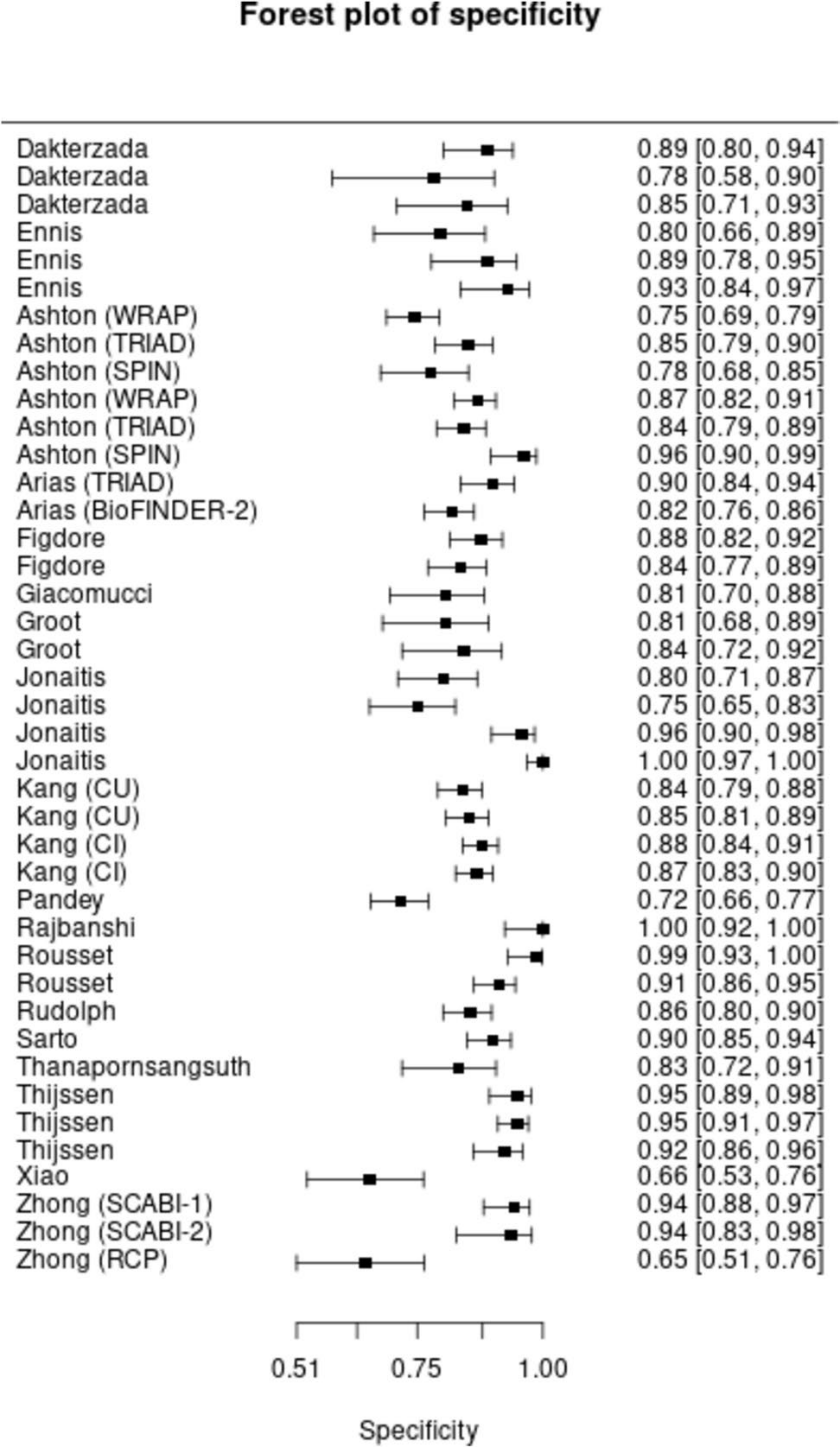
Forest Plot showing the Specificity of p-tau217 in diagnosing AD

Across the included studies, plasma p-tau217 showed high overall accuracy for identifying AD defined by biomarker positivity (amyloid and/or tau) (Fig. 2). The Bayesian bivariate (HSROC) model estimated a pooled sensitivity of 85.4% (95% posterior interval [PI]: 81.4–88.7) (Fig. 3) and specificity of 88.0% (PI: 85.1–90.6) (Fig. 4), with PLR 7.13 (5.8–9.0), NLR 0.167 (0.129–0.210), and DOR 42.72 (31.7–60.9). Between-study heterogeneity on the logit scale was marked (standard deviation [SD] of sensitivity = 0.950 [PI: 0.755–1.218]; SD of specificity = 0.758 [PI: 0.557–1.040]) and the sensitivity–specificity correlation was negative (r = −0.464, PI = −0.713 to −0.120), a pattern consistent with threshold effects. In meta-regression using study-level cut-off as a covariate, neither the shape (β = −0.224, PI = −0.591 to 0.154) nor mean cutpoint (β = −0.329, PI = −0.735 to 0.069) parameters showed clear evidence of a shift in HSROC location/shape (credible intervals crossed zero). Posterior SDs for the cut point (0.249, PI = 0.070–0.643) and accuracy component (0.998, 0.282–2.573) indicate meaningful threshold variability across studies, but no strong evidence that average cut-off alone explains between-study differences.

**Fig. 5:**
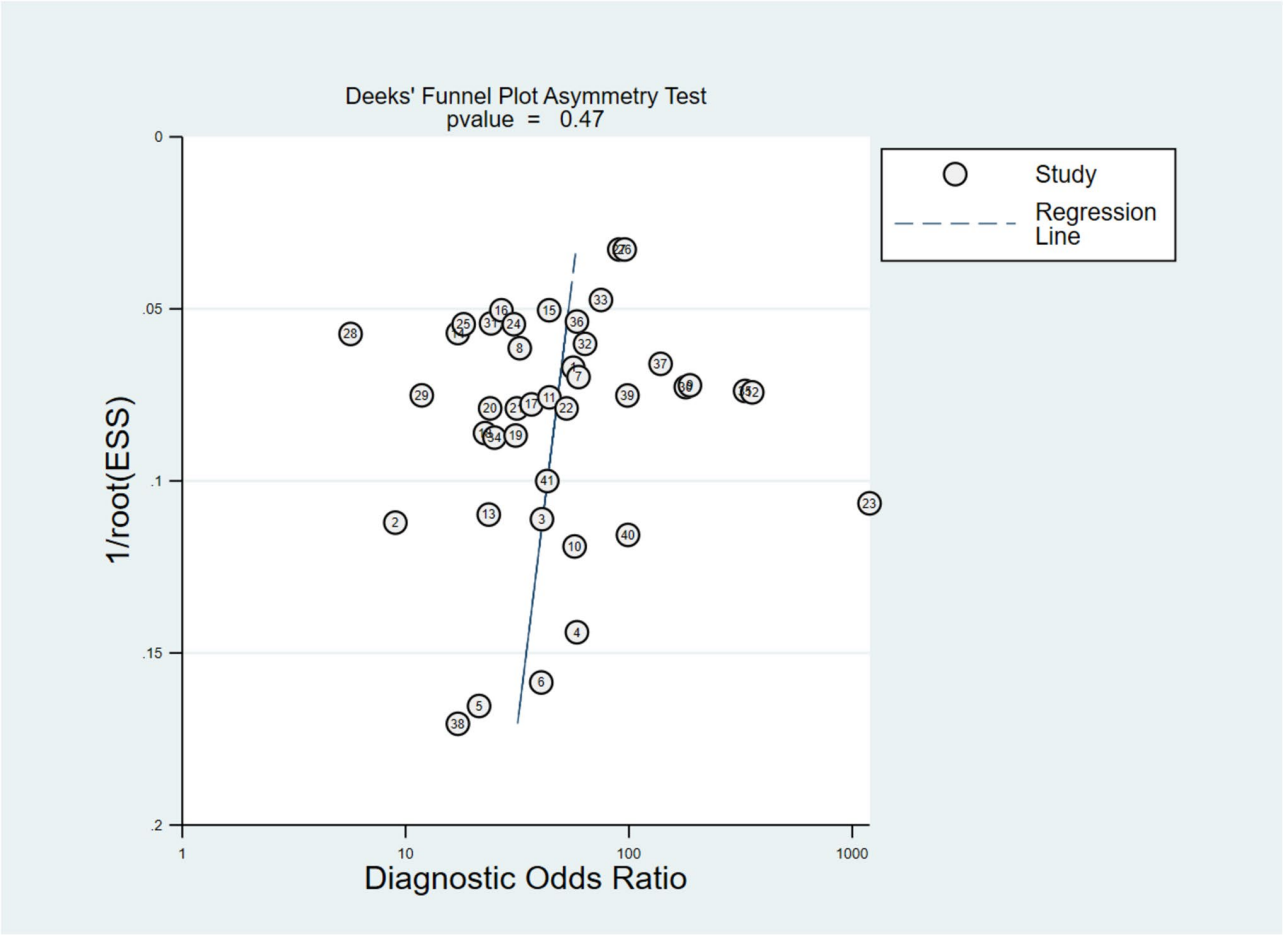
Deek’s Funnel plot for evaluation of publication bias

**Fig. 6:**
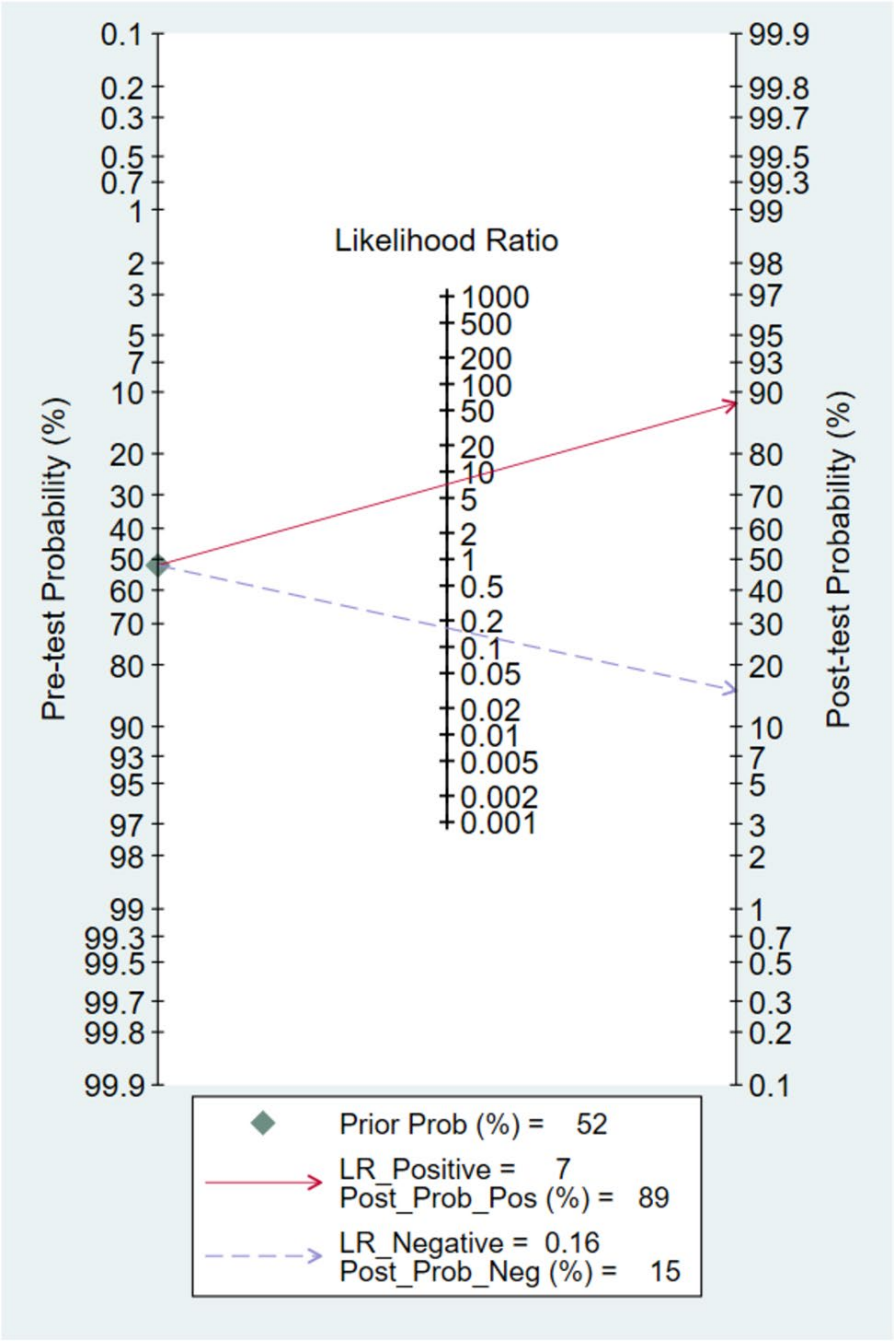
Fagan’s Nomogram for clinical utility

In assessment of small-study effects, Deeks’ funnel plot showed no evidence of asymmetry (p = 0.47), with study points reasonably symmetric around the regression line across the range of diagnostic odds ratios—arguing against important publication bias (Fig. 5). To translate the pooled likelihood ratios to practice, the Fagan nomogram illustrates that, at an illustrative pre-test probability of 50%, a positive p-tau217 result (PLR = 7.13) raises the post-test probability to 89%, whereas a negative result (NLR = 0.167) lowers it to 15% (Fig. 6). These shifts are consistent with moderate-to-large diagnostic effects: positives effectively “rule in” AD-consistent biomarker positivity and justify confirmatory PET/CSF, while negatives substantially reduce but do not eliminate probability when baseline risk is high, supporting p-tau217 as a triage tool with cut-offs tuned to local priorities (rule-in vs rule-out).

#### Subgroup Analyses by Reference Standard (Amyloid+, Tau+, Clinical AD)

In these analyses, amyloid+ and tau+ indicate AD pathology defined by positive amyloid or tau biomarkers (PET or CSF, using study-specific cut-offs), whereas ‘clinical AD’ indicates a clinical diagnosis of AD dementia based on established criteria without biomarker confirmation.

Performance was robust across targets (Figures S3, and S4). For amyloid positivity, pooled sensitivity was 87.3% (95% PI: 84.7–89.5) and specificity 85.5% (PI: 83.1–87.7), yielding PLR 6.03 (PI: 5.16–7.09), NLR 0.15 (PI: 0.12–0.18), and DOR 40.90 (PI: 30.74–52.51). Heterogeneity was modest (SD of sensitivity = 0.469 [PI: 0.351–0.625]; SD of specificity 0.417 [PI: 0.295–0.573]) with a weak, non-significant negative correlation (r = −0.167, PI: −0.380 to 0.072). For tau positivity, sensitivity and specificity were 84.9% (PI: 72.8–91.8) and 93.8% (PI: 86.3–97.0), with PLR 13.61 (PI: 5.90–28.23), NLR 0.16 (PI: 0.09–0.29), and DOR 86.36 (PI: 25.82–239.94); heterogeneity was moderate and the correlation imprecisely positive (r = 0.121, PI: −0.362 to 0.596). For clinically diagnosed AD, sensitivity was 72.9% (PI: 55.8–84.8) and specificity 89.5% (PI: 82.4–94.1), giving PLR 6.90 (PI: 3.93–12.20), NLR 0.30 (PI: 0.17–0.49), and DOR 23.20 (PI: 9.43–57.11); heterogeneity was larger (SD of sensitivity = 1.005 [PI: 0.747–1.356]; SD specificity = 0.755 [PI: 0.473–1.143]) with a negative, non-significant correlation (r = −0.151, PI: −0.480 to 0.191).

### Subgroup Analyses of Amyloid Positivity by Modality

Amyloid positivity was defined using amyloid PET or CSF Aβ measures (e.g., Aβ42 or Aβ42/40 ratio) according to study-specific thresholds (PET vs CSF).

When restricted to amyloid positivity and stratified by the reference, accuracy was comparable (Figures S5, and S6). Against amyloid PET, pooled sensitivity was 86.4% (95% PI 82.8–89.4) and specificity 85.9% (PI: 82.4–88.9), with PLR 6.14 (PI: 4.91–7.70), NLR 0.16 (PI: 0.12–0.20), and DOR 38.64 (PI: 27.42–54.45). Against CSF-based reference, sensitivity was 88.0% (PI: 83.1–92.0) and specificity 84.4% (PI: 79.2–88.6), with PLR 5.65 (PI: 4.25–7.67), NLR 0.14 (PI: 0.09–0.20), and DOR 40.29 (PI: 23.95–70.34). Heterogeneity within each subgroup was mild and broadly similar, and the overlapping PIs indicate no performance difference by reference standard.

Within the amyloid-positivity analysis, Deeks’ funnel plot indicated small-study effects (p = 0.05, significant at α = 0.10), with a positive regression slope suggesting that smaller studies tended to report larger diagnostic odds ratios (Figure S7). This implies a possible publication/size bias and supports cautious interpretation of the pooled accuracy. Translating the model to practice, the Fagan nomogram shows that at a representative pre-test probability of 50%, a positive p-tau217 result (PLR = 6) increases the post-test probability to 88%, whereas a negative result (NLR = 0.14) reduces it to 14% (Figure S8). These probability shifts remain clinically meaningful for triage to confirmatory PET/CSF, while the observed asymmetry underscores the value of sensitivity analyses.

#### Qualitative Synthesis

### Diagnostic Specificity and Accuracy

Across multiple cohorts, plasma p-tau217 consistently outperformed other biomarkers (Aβ42/40, t-tau, NfL, and even p-tau181) in distinguishing AD from controls and non-AD dementias. Li et al. [35] reported area under curve (AUCs) up to 0.983 for differentiating AD from controls and 0.943 for MCI vs cognitively unimpaired, with p-tau217 correlating strongly with memory performance. Brickman et al. [24] found that lower Aβ42/40 and higher p-tau217 predicted incident AD diagnoses and correlated with both clinical and autopsy findings. Palmqvist et al. [37] validated an automated Lumipulse assay, with diagnostic accuracy of AUC 0.93–0.96, high concordance with amyloid PET (> 90%) and tau PET (83%).

### Prognostic Value and Longitudinal Prediction

Aside from diagnosis, p-tau217 also reveals imminent progression of disease. Moon et al. [36] demonstrated that baseline plasma p-tau217 was a predictor of subsequent tau-PET accumulation in cortical regions in preclinical AD and hence p-tau217 can be utilized as a trajectory marker. Brickman et al. [24] also reported that plasma p-tau217 was a predictor of subsequent clinical onset of AD.

### Cognitive Domain Associations

Sewell et al. demonstrated higher plasma p-tau217 was only associated with worse episodic memory, showing that p-tau217 detects AD-related cognitive impairment. Li et al. [35] validated memory impairment associations again, building further evidence on p-tau217 specificity for typical AD impairments. Lastly, Wang et al. [48] demonstrated that in Aβ-positive individuals, homocysteine and p-tau217 co-operated to hasten temporal cortical thinning and cognitive impairment. This indicates that modifiable metabolic hazards can enhance AD pathology detectable by p-tau217.

### Behavioral and Frailty Correlates

Ghahremani et al. [30] demonstrated that mild behavioral impairment was associated with increased plasma p-tau217 and threefold increased likelihood of biomarker positivity, even in adults’ dementia-free. Tian et al. [47] associated physical frailty with increased p-tau217, GFAP, and NfL, and decreased Aβ42/40, with frailty which had an AUC = 0.83 to discriminate between dementia and non-dementia. Both studies show clinical phenotypes (e.g., frailty, and behavioral change) can label people with underlying AD biology.

## Discussion

AD gradually robs people of their ability to remember and think, making even the simplest of tasks more challenging [51]. AD is characterized by specific changes in the brain, namely the build-up of plaque composed of sticky amyloid-b proteins, and twisted tau proteins that create disruption to the normal exchange of information between nerve cells [52, 53]. Traditionally, clinicians have relied on invasive tests like lumbar punctures to collect spinal fluid or costly brain imaging scans to identify these changes [54–56]. In recent times, though, scientists have been investigating blood samples as a less invasive and more accessible way to detect the signs of Alzheimer’s [57–60]. Among these blood-based indicators, plasma phosphorylated tau at position 217, known as p tau217, has emerged as a particularly promising marker because it reflects the tau-related brain changes characteristic of the disease [14, 61, 62]. While several studies have suggested this marker can help not only to diagnose AD but also to keep track of its progression, there are still some questions to answer [14, 61]. Lab methods for measuring p-tau217 aren’t fully standardized, different patient groups vary widely, and researchers have used a range of ‘gold standards’ like brain scans and fluid measures to confirm diagnoses.

Among the 27 studies reviewed, plasma p-tau217 emerged as a notably dependable marker for identifying AD, outperforming traditional methods that detect amyloid and tau alterations in the brain. On average, the test successfully detected AD-related brain changes in approximately 85% of individuals who had the condition, while correctly excluding about 88% of those without such changes. This equilibrium indicates that the test is both highly sensitive to the presence of disease and sufficiently specific to minimizing false positives. Its diagnostic effectiveness was further highlighted by a strong odds ratio, underlining its ability to differentiate between affected and unaffected individuals with great accuracy. The reliability of p-tau217 held steady across participants regardless of cognitive status from those with intact cognition to individuals experiencing mild cognitive impairment or advanced dementia. This marker demonstrated solid diagnostic performance whether the comparison was made against tests for amyloid or tau brain pathology, or even clinical diagnosis based solely on symptoms without biomarker confirmation. However, because clinical diagnosis can misclassify underlying AD pathology, results using clinical diagnosis as the reference standard should be interpreted cautiously. Additionally, p-tau217 showed comparable accuracy when measured against brain imaging or CSF assays. The consistent findings spanning nearly 20,000 subjects across diverse research designs indicate that p-tau217 holds promise as a versatile and practical blood-based biomarker for assisting clinicians in the early detection and management of AD.

Among those with amyloid, our combined data revealed that the blood test identified roughly 87 out of 100 actual cases and accurately eliminated roughly 85 out of 100 without disease. These figures closely match what previous research had determined in comparing plasma p-tau217 with brain imaging and spinal tests [34, 39, 41]. Interestingly, the test was even better at identifying tau-related brain changes, with an accuracy of over 90%, supporting the idea that p-tau217 is closely tied to the tau-related damage seen in AD. Similar findings were reported by Arias et al. and Ashton et al., further confirming this blood marker’s link to tau pathology. Diagnostic accuracy for clinically diagnosed AD was slightly lower (sensitivity 72.9%), as might be expected given that clinical diagnosis is more modestly correlated with underlying pathology [27]. These patterns confirm plasma p-tau217 as a loyal surrogate for biomarker-confirmed AD pathology but less definitive without biomarker confirmation.

The comparable diagnostic accuracy of plasma p-tau217 against amyloid PET and CSF supports its role as a practical and less invasive alternative to conventional biomarker assays. Published studies similarly demonstrate high agreement between plasma p-tau217 and established PET/CSF markers [25, 28, 33]. The slight indication of small-study effects in amyloid PET subgroup highlights the need for continued large-scale validation, reflecting known publication bias concerns in biomarker research [28].

A similar meta-analysis was conducted before by Khalafi et al. [63], but some differences exist between ours and theirs. Although both studies evaluate p-tau217, they address distinct research questions using different inclusion criteria, reference standards, and analytical frameworks, resulting in complementary rather than redundant findings. First, the scope and biomarker focus differ fundamentally. Khalafi et al. [63] examined both plasma and CSF p-tau217, whereas the present meta-analysis is exclusively focused on plasma p-tau217. This deliberate restriction was chosen to evaluate plasma p-tau217 as a stand-alone, minimally invasive diagnostic biomarker, which has direct implications for large-scale screening, real-world clinical implementation, and trial prescreening. By excluding CSF-based studies, our analysis avoids conflating invasive and non-invasive modalities and provides plasma-specific diagnostic estimates that are not directly extractable from the Khalafi et al. [63] meta-analysis. Second, the definition of the target condition and reference standards differs. Khalafi et al. [63] restricted their reference standards to amyloid-PET and tau-PET positivity, thereby addressing the accuracy of p-tau217 for detecting imaging-defined AD pathology. In contrast, our study adopts a broader and clinically oriented framework, including amyloid PET, tau PET, CSF biomarker-defined AD, and when biomarker confirmation was unavailable, established clinical diagnostic criteria (e.g., NIA-AA). This approach reflects real-world diagnostic pathways and allows evaluation of plasma p-tau217 across heterogeneous clinical and biomarker settings, which were not examined in the prior study. Third, the analytical strategy and outcomes synthesized are different. Khalafi et al. [63] primarily reported pooled sensitivity and specificity stratified by PET modality and cognitive status. In contrast, we performed a formal diagnostic test accuracy meta-analysis, synthesizing sensitivity, specificity, positive and negative likelihood ratios, DORs, and summary receiver operating characteristic (SROC) curves using a bivariate model. This provides a single, clinically interpretable measure of discrimination (DOR) and enables assessment of overall test performance across varying diagnostic thresholds analyses not reported in the earlier work. The inclusion of a Fagan nomogram represents an important translational component of this meta-analysis, linking statistical measures of diagnostic accuracy to clinically meaningful decision-making. While pooled sensitivity, specificity, and summary ROC curves describe test performance at the population level, they do not directly convey how a test result alters the probability of disease in an individual patient. The Fagan nomogram addresses this gap by integrating pre-test probability with pooled likelihood ratios to estimate post-test probability. In the present meta-analysis, the Fagan nomogram demonstrates how plasma p-tau217 meaningfully shifts the probability of AD pathology following either a positive or negative test result. By applying pooled positive and negative likelihood ratios derived from the bivariate meta-analysis, the nomogram provides an intuitive visualization of the diagnostic impact of plasma p-tau217 across clinically plausible pre-test probabilities. This allows clinicians to appreciate not only whether the biomarker is statistically accurate, but also whether it produces a clinically relevant change in diagnostic certainty. Importantly, the Fagan nomogram facilitates assessment of plasma p-tau217 in different clinical contexts, such as memory clinics with high pre-test probability and screening or prescreening settings with lower baseline prevalence. This highlights the potential role of plasma p-tau217 as a rule-in or rule-out test depending on the clinical scenario, an aspect that cannot be inferred from sensitivity and specificity alone. Fourth, study inclusion and evidence base differ. Our literature search extends through July 2025, capturing multiple recently published plasma p-tau217 studies that were unavailable to Khalafi et al. [63], whose search concluded in August 2024. Moreover, Khalafi et al. [63] excluded studies lacking PET-based diagnostic accuracy data, whereas we included additional plasma p-tau217 studies reporting validated diagnostic outcomes against CSF biomarkers or clinical diagnosis, further expanding the evidence base relevant to routine practice. Finally, heterogeneity and subgroup analyses were addressed differently. While Khalafi et al. [63] explored heterogeneity primarily in relation to PET modality and assay platform, our study further investigates heterogeneity using subgroups and meta-regression according to reference standard type, diagnostic definition, and clinical versus biomarker-confirmed AD. This provides additional insight into sources of variability and conditions under which plasma p-tau217 performs optimally.

Recent research by Tian and colleagues [47] demonstrated notable links between two frailty types biopsychological frailty and physical frailty and several cognitive disorders, including dementia, AD, vascular dementia, and mild cognitive impairment. Among these frailty profiles, physical frailty showed particular connections with plasma markers associated with AD’s pathology, such as lowered Aβ42/Aβ40 ratios and elevated p-tau217 levels. Interestingly, physical frailty appeared more strongly correlated with vascular dementia compared to AD. In a related study, Wang et al. [48] found that p-tau217 independently and interactively links to thinning in the temporal cortex and associated cognitive impairments. Meanwhile, Sewell et al. [44] reported that increased p-tau217 specifically corresponded with declines in episodic memory and could aid in distinguishing cognitive impairments related to AD from those caused by other factors, revealing underlying heterogeneity in aging-related cognitive decline.

Palmqvist et al. observed that while p-tau217’s diagnostic accuracy diminished somewhat in secondary care settings, it remained unaffected by chronic kidney disease, diabetes, sex, APOE genotype, or cognitive stage. Brickman et al. [24] supported these findings, highlighting that concentrations of p-tau181 and p-tau217 were elevated in both clinically and pathologically confirmed AD cases, and that a reduced Aβ42/Aβ40 ratio alongside increased p-tau levels predicted subsequent AD diagnosis. Furthermore, Ghahremani et al. [30] linked mild behavioral impairment (MBI) an early indicator of AD risk to higher plasma p-tau217 levels, showing that MBI raised the likelihood of p-tau217 positivity even in individuals without dementia.

Moon and colleagues [36] found p-tau217 to align with amyloid-beta presence broadly across cortical areas, as well as with tau accumulation in temporo-parietal regions. Their longitudinal study identified that high p-tau217 levels predicted global tau burden in the brain during preclinical AD phases, but not with accompanying amyloid-beta alteration. Li et al. [35] further emphasized plasma p-tau217’s superior performance compared to other markers in differentiating AD across varied populations. Complementing this, Saari et al. [42] noted that p-tau217 levels tend to increase with age, with nearly 39% of individuals demonstrating abnormal levels above 0.42 pg/mL; however, factors like sex, education, and AD polygenic risk scores did not show an association with p-tau217.

Recent international recommendations have further clarified the role of blood-based biomarkers in AD. The International Working Group (IWG) and Alzheimer’s Association (AAIC) emphasize that blood biomarkers, including plasma p-tau217, should currently be used as triage tools rather than standalone diagnostic tests, particularly in clinical practice. The revised criteria highlight the importance of confirming pathology using established biomarkers (PET or CSF), while recognizing the growing role of plasma biomarkers in screening, risk stratification, and clinical trial recruitment [9, 59, 64, 65]. Our findings align with these recommendations, supporting plasma p-tau217 as a high-performing triage biomarker that can guide confirmatory testing.

### Clinical Implications

Given its high accuracy, plasma p-tau217 could be deployed as a frontline screening tool to triage patients for more invasive and costly biomarker testing such as PET imaging or CSF analysis. Its capacity to both rule in and rule out pathology enhances clinical decision-making, particularly in primary care and memory clinic settings. Moreover, plasma p-tau217’s non-invasiveness and potential for repeat sampling support its integration into longitudinal monitoring and therapeutic trials targeting AD pathology [66].

### Future Research Directions

Further research should aim to standardize assay methods and establish universally accepted cut-off thresholds to reduce inter-study variability. Longitudinal studies examining plasma p-tau217 trajectories in preclinical and prodromal AD stages are critical for early diagnosis and monitoring disease progression. Additionally, studies in underrepresented populations and real-world clinical settings would improve understanding of biomarker utility across diverse demographic and clinical contexts.

### Strengths and Limitations

The strengths of our meta-analysis include the large, pooled sample size, inclusion of diverse clinical populations, rigorous bias assessment, and stratified subgroup analyses. However, limitations include heterogeneity in assay platforms and cut-off values contributing to variability, potential residual publication bias especially in smaller studies, and a preponderance of data from primarily memory clinic cohorts which may limit generalizability to broader populations. Additionally, reference standards differed across studies and a minority relied on clinical diagnosis without biomarker or neuropathological confirmation; this is prone to misclassification and likely contributed to the lower sensitivity and higher heterogeneity observed in the clinical AD subgroup. Accordingly, our primary conclusions emphasize biomarker-defined reference standards (amyloid and/or tau positivity by PET/CSF). The borderline asymmetry observed in the amyloid subgroup (p = 0.05) suggests potential small-study effects rather than definitive publication bias. This may reflect a tendency for smaller studies to report inflated diagnostic accuracy, which has been described in biomarker research. However, the interpretation of Deeks’ test should remain cautious, as its statistical power is limited, particularly when the number of included studies is modest and heterogeneity is substantial.

## Conclusion

This meta-analysis indicates that plasma p-tau217 demonstrates promising diagnostic accuracy for detecting AD pathology across biomarker-defined reference standards. However, heterogeneity across assays, populations, and reference definitions, along with the use of optimized cut-offs in some studies and the limited power of publication-bias assessments, warrant cautious interpretation. Plasma p-tau217 appears well suited as a triage biomarker to guide confirmatory testing, but further large, prospectively designed studies with standardized assays and externally validated thresholds are needed before widespread clinical implementation.

## Supplementary Information

Below is the link to the electronic supplementary material.

Supplementary file1 (PNG 1390 KB)

Supplementary file2 (PNG 87 KB)

Supplementary file3 (PNG 50 KB)

Supplementary file4 (PNG 23 KB)

Supplementary file5 (PNG 44 KB)

Supplementary file6 (PNG 18 KB)

Supplementary file7 (PNG 70 KB)

Supplementary file8 (PNG 77 KB)

Supplementary file9 (DOCX 17 KB)

## Data Availability

All data used in this manuscript are present in the original file.
