# Supplementary figures and images for: From Cerebrospinal Fluid to Blood Draw: Plasma p-Tau217 as a Non-Invasive Biomarker for Alzheimer’s Disease: A Fagan Nomogram-Based Meta-Analytic Study
Source: Mol Neurobiol. 2026 May 4;63(1):606. doi: 10.1007/s12035-026-05864-2 (PMC13136221; doi:10.1007/s12035-026-05864-2)

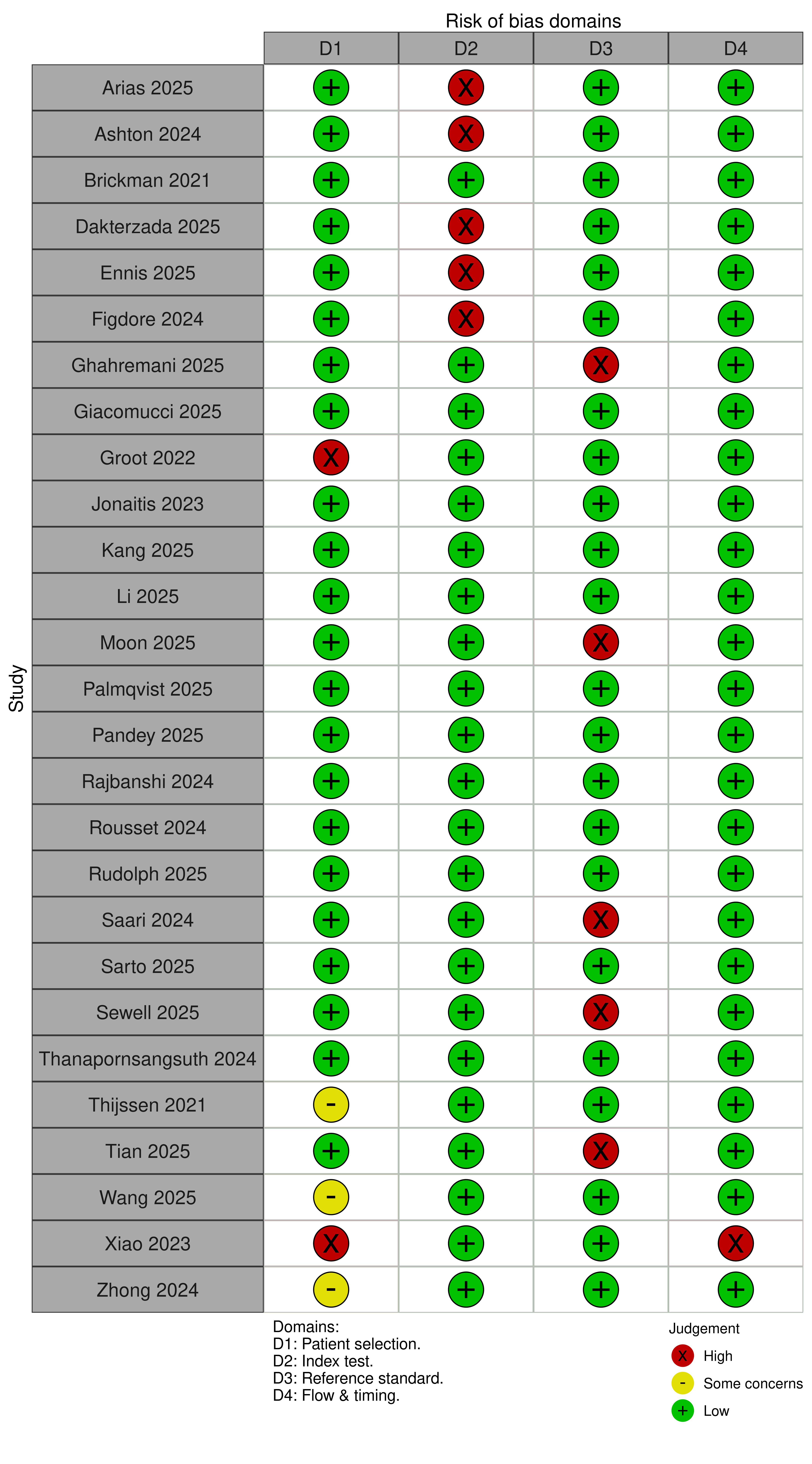

Supplement: Supplementary file 1 — Supplementary file1 (PNG 1390 KB) [file 12035_2026_5864_MOESM1_ESM.png]

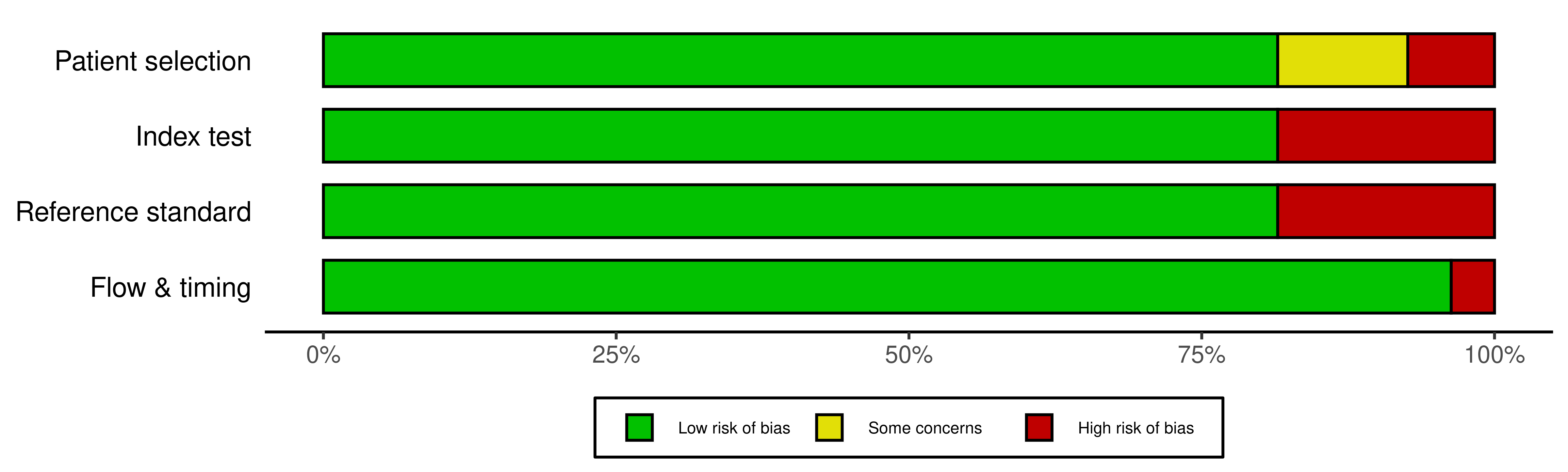

Supplement: Supplementary file 2 — Supplementary file2 (PNG 87 KB) [file 12035_2026_5864_MOESM2_ESM.png]

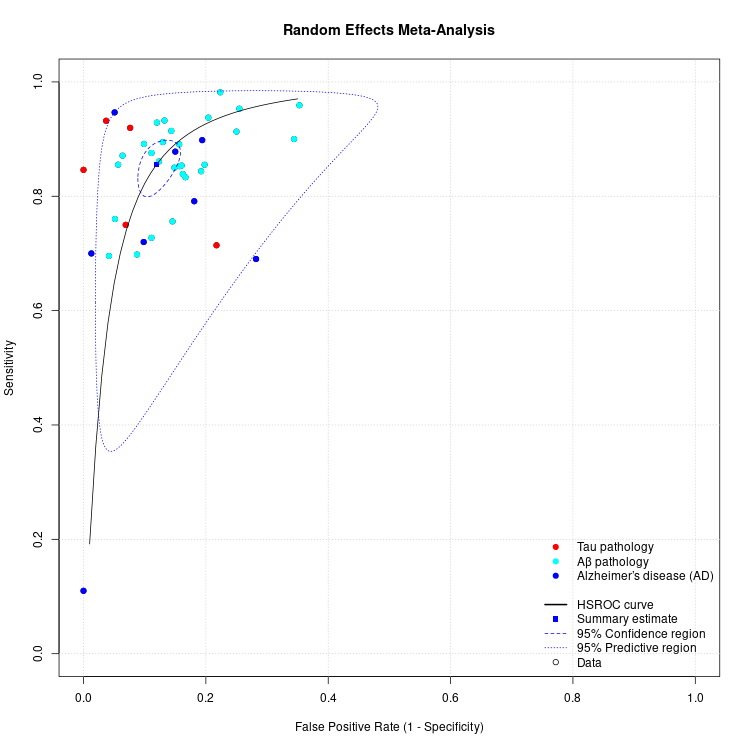

Supplement: Supplementary file 3 — Supplementary file3 (PNG 50 KB) [file 12035_2026_5864_MOESM3_ESM.png]

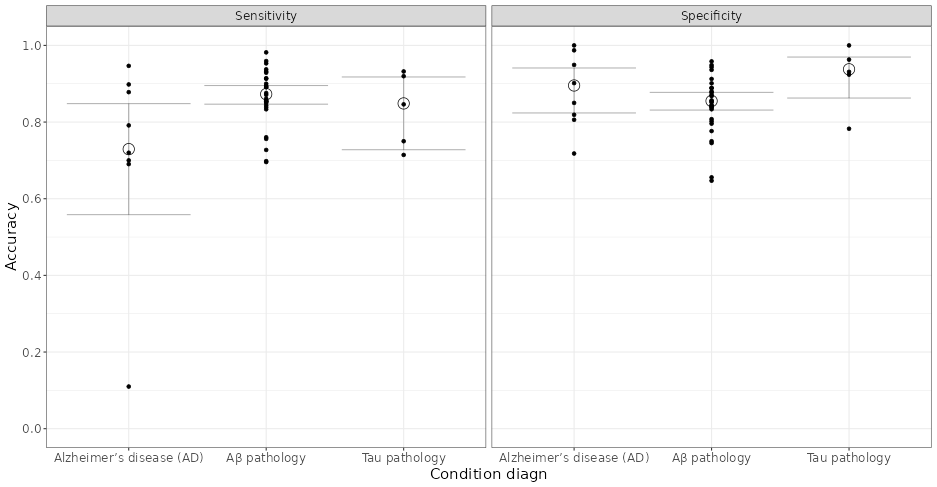

Supplement: Supplementary file 4 — Supplementary file4 (PNG 23 KB) [file 12035_2026_5864_MOESM4_ESM.png]

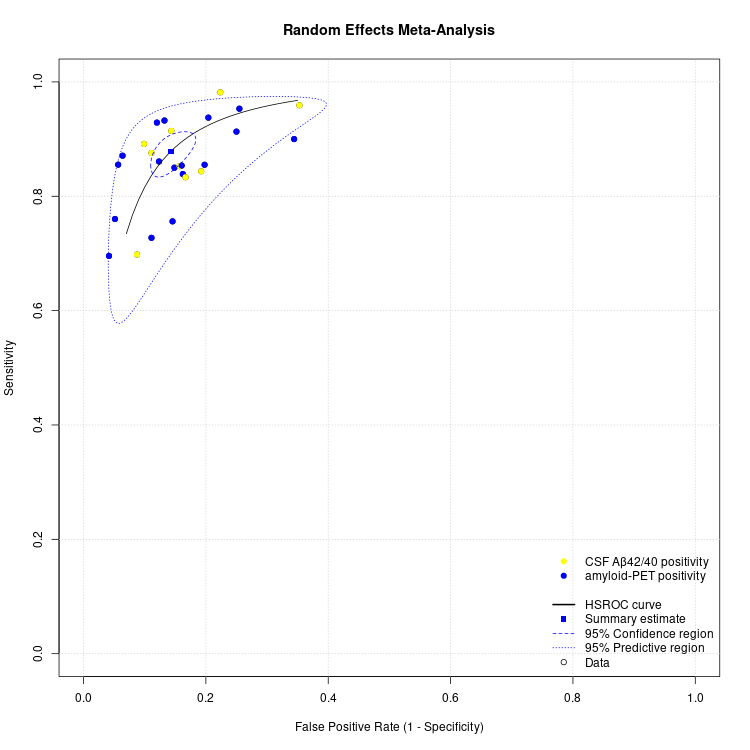

Supplement: Supplementary file 5 — Supplementary file5 (PNG 44 KB) [file 12035_2026_5864_MOESM5_ESM.png]

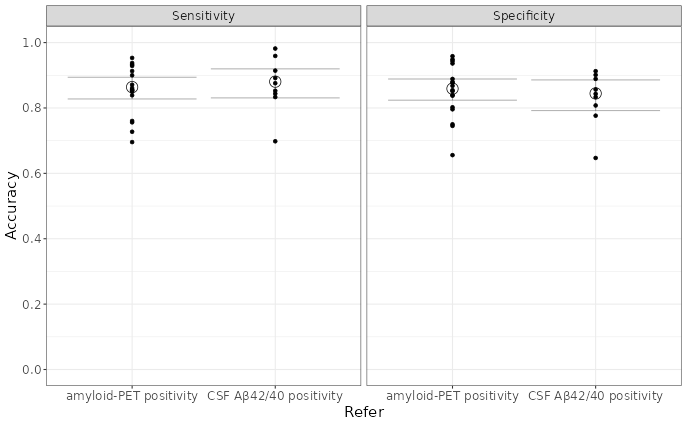

Supplement: Supplementary file 6 — Supplementary file6 (PNG 18 KB) [file 12035_2026_5864_MOESM6_ESM.png]

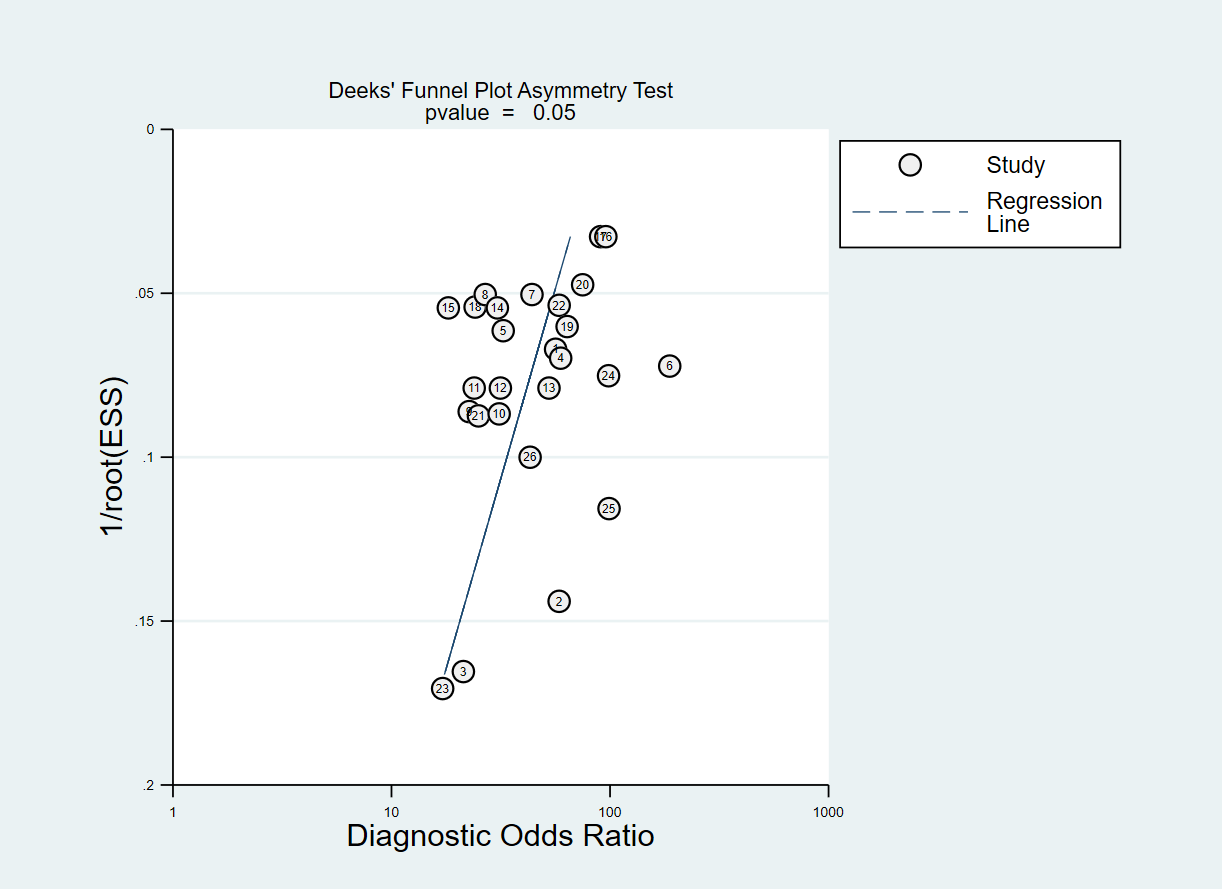

Supplement: Supplementary file 7 — Supplementary file7 (PNG 70 KB) [file 12035_2026_5864_MOESM7_ESM.png]

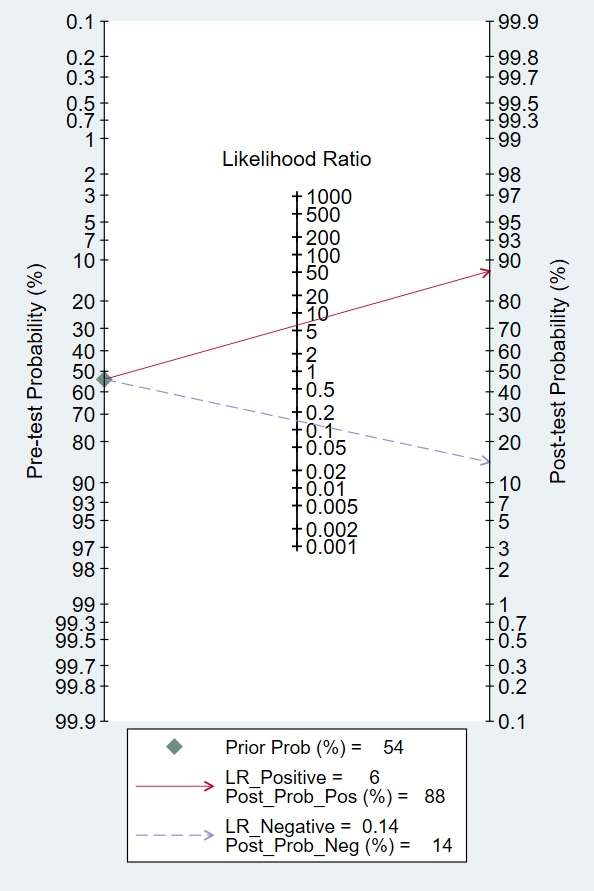

Supplement: Supplementary file 8 — Supplementary file8 (PNG 77 KB) [file 12035_2026_5864_MOESM8_ESM.png]
