# Supplementary material for: From Cerebrospinal Fluid to Blood Draw: Plasma p-Tau217 as a Non-Invasive Biomarker for Alzheimer’s Disease: A Fagan Nomogram-Based Meta-Analytic Study
Source: Mol Neurobiol. 2026 May 4;63(1):606. doi: 10.1007/s12035-026-05864-2 (PMC13136221; doi:10.1007/s12035-026-05864-2)
Supplement: Supplementary file 9 — Supplementary file9 (DOCX 17 KB) [file 12035_2026_5864_MOESM9_ESM.docx]

**Table S1: Tabular presentation for QUADAS-2 results**

| **Study** | **RISK OF BIAS** | | | | **APPLICABILITY CONCERNS** | | |
| --- | --- | --- | --- | --- | --- | --- | --- |
|  | **PATIENT SELECTION** | **INDEX TEST** | **REFERENCE STANDARD** | **FLOW AND TIMING** | **PATIENT SELECTION** | **INDEX TEST** | **REFERENCE STANDARD** |
| Arias 2025 | ☺ | ☹ | ☺ | ☺ | ☺ | ☺ | ☺ |
| Ashton 2024 | ☺ | ☹ | ☺ | ☺ | ☺ | ☺ | ☺ |
| Brickman 2021 | ☺ | ☺ | ☺ | ☺ | ☺ | ☺ | ☺ |
| Dakterzada 2025 | ☺ | ☹ | ☺ | ☺ | ☺ | ☺ | ☺ |
| Ennis 2025 | ☺ | ☹ | ☺ | ☺ | ☺ | ☺ | ☺ |
| Figdore 2024 | ☺ | ☹ | ☺ | ☺ | ☹ | ☺ | ☺ |
| Ghahremani 2025 | ☺ | ☺ | ☹ | ☺ | ☺ | ☺ | ☹ |
| Giacomucci 2025 | ☺ | ☺ | ☺ | ☺ | ☺ | ☺ | ☺ |
| Groot 2022 | ☹ | ☺ | ☺ | ☺ | ☹ | ☺ | ☺ |
| Jonaitis 2023 | ☺ | ☺ | ☺ | ☺ | ☺ | ☺ | ☺ |
| Kang 2025 | ☺ | ☺ | ☺ | ☺ | ☺ | ☺ | ☺ |
| Li 2025 | ☺ | ☺ | ☺ | ☺ | ☺ | ☺ | ☺ |
| Moon 2025 | ☺ | ☺ | ☹ | ☺ | ☺ | ☺ | ☹ |
| Palmqvist 2025 | ☺ | ☺ | ☺ | ☺ | ☺ | ☺ | ☺ |
| Pandey 2025 | ☺ | ☺ | ☺ | ☺ | ☺ | ☺ | ☺ |
| Rajbanshi 2024 | ☺ | ☺ | ☺ | ☺ | ☺ | ☺ | ☺ |
| Rousset 2024 | ☺ | ☺ | ☺ | ☺ | ☺ | ☺ | ☺ |
| Rudolph 2025 | ☺ | ☺ | ☺ | ☺ | ☺ | ☺ | ☺ |
| Saari 2024 | ☺ | ☺ | ☹ | ☺ | ☺ | ☺ | ☹ |
| Sarto 2025 | ☺ | ☺ | ☺ | ☺ | ☺ | ☺ | ☺ |
| Sewell 2025 | ☺ | ☺ | ☺ | ☺ | ☺ | ☺ | ☹ |
| Thanapornsangsuth 2024 | ☺ | ☺ | ☺ | ☺ | ☺ | ☺ | ☺ |
| Thijssen 2021 | ? | ☺ | ☺ | ☺ | ☺ | ☺ | ☺ |
| Tian 2025 | ☺ | ☺ | ☹ | ☺ | ☺ | ☺ | ☹ |
| Wang 2025 | ? | ☺ | ☺ | ☺ | ? | ☺ | ☺ |
| Xiao 2023 | ☹ | ☺ | ☺ | ☹ | ☹ | ☺ | ☺ |
| Zhong 2024 | ? | ☺ | ☺ | ☺ | ? | ☺ | ☺ |

☺Low Risk ☹High Risk ? Unclear Risk
